# Supplementary material for: Mechanochemistry and Eco-Bases for Sustainable Michael Addition Reactions
Source: Molecules. 2022 May 21;27(10):3306. doi: 10.3390/molecules27103306 (PMC9144101; doi:10.3390/molecules27103306)
Supplement: Supplementary file 1 [file molecules-27-03306-s001.zip › molecules-1712237-supplementary.pdf]

molecules
MDPI

Supporting information

Mechanochemistry and Eco-bases for Sustainable Michael Addition Reactions

Yvette Lock Toy Ki [1], Armelle Garcia [1], Franck Pelissier [1], Tomasz K. Olszewski [2], Alicja Babst-Kostecka [3], Yves-Marie Legrand [1] and Claude Grison [1,*]

1. Laboratory of Bio-inspired Chemistry and Ecological Innovations (ChimEco), UMR 5021, CNRS, University of Montpellier, Cap Delta, 1682 rue de la Valsière, 34790 Grabels, France; yvette.lock-toy-ki@cnrs.fr (Y.L.T.K.); armelle.garcia@cnrs.fr (A.G.); franck.pelissier@cnrs.fr (F.P.); yves-marie.legrand@umontpellier.fr (Y.-M.L.)
2. Department of Physical and Quantum Chemistry, Faculty of Chemistry, Wroclaw University of Science and Technology, Wybrzeze Wyspianskiego 29, 50-370 Wroclaw, Poland; tomasz.olszewski@pwr.edu.pl
3. Department of Environmental Science, The University of Arizona, 1110 E. South Campus Drive, Saguaro Hall, Building 33, Tucson, AZ 85721, USA; ababstkostecka@arizona.edu

* Correspondence: claude.grison@cnrs.fr

1. Spectral data

1.1. Dimethyl 2-(3-oxocyclopentyl)malonate

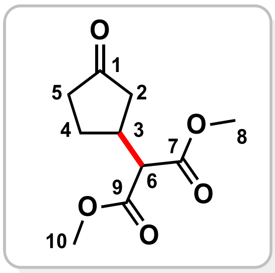

^{1}H NMR (400 MHz, CDCl₃) δ (ppm): 3.75 (1 H, s, **H-8**), 3.73 (1H, s, **H-10**), 3.37 (1H, d, *J* = 9.4 Hz, **H-6**), 2.88-2.80 (1 H, m, **H-3**), 2.49 (1H, dd, *J* = 18.4, 7.6 Hz, **H-2a**), 2.38-2.2 (1 H, m, **H-6a**), 2.27-2.22 (1H, m, **H-2b**), 2.22-2.15 (1H, m, **H-6b**) 1.99 (1H, ddd, *J* = 18.4, 11.0, 1.2 Hz, **H-4a**), 1.71-1.57 (1H, m, **H4-b**)

^{13}C NMR (100 MHz, CDCl₃) δ (ppm): 217.0 (**C-1**), 168.64 (**C-7**), 168.5 (**C-9**), 56.2, (**C-6**), 52.8 (x2) (**C-8, C-10**), 43.0 (**C-3**), 38.3 (**C-2**), 36.5 (**C-6**), 27.6 (**C-4**).

IR (neat, cm^{-1}) : 2959, 2921, 2852, 1729,1436, 1147.

1.2.Dimethyl 2-(3-oxocyclohexyl)malonate

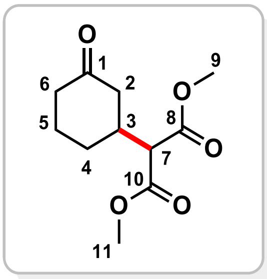

^{1}H NMR (400 MHz, CDCl₃) δ (ppm): 3.72 (3H, s, **H-9**), 3.71 (3H, s, **H-11**), 3.32 (1H, d, *J* = 8 Hz, **H-7**), 2.51 (1H, dddd, *J* = 19.4, 15.7, 11.8, 7.7, 3.8 Hz, **H-3**), 2.39-2.45 (2H, m, **H-6a, H-2b**), 2.23-2.29 (2H, m, **H-6b, H-2b**), 2.04 (1H, dddd, *J* = 14.6, 10.4, 6.9, 3.6, **H-5a**), 1.93-1.96 (m, 1H, **H-4a**), 1.64-1.73 (1H, m, **H-5b**), 1.46-1.55 (1H, m, **H-4b**).

^{13}C NMR (100 MHz, CDCl₃) δ (ppm): 209.6 (**C-1**), 168.3 (**C-8, C-10**), 56.7 (**C-7**), 52.7 (x2) (**C-9, C-11**), 45.1 (**C-2**), 41.1 (**C-6**), 38.2 (**C-3**), 28.9 (**C-5**), 24.6 (**C-4**).

IR (neat, cm^{-1}) : 2955, 2868, 1732, 1710, 1435, 1150.

1.3. Dimethyl 2-(3-oxo-1,3-diphenylpropyl)malonate

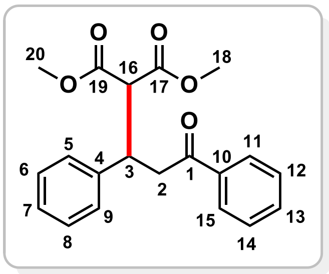

^{1}H NMR (400 MHz, $CDCl_3$) δ (ppm): 7.90 (2H, ddd, *J* = 8.1, 2.9, 1.8, **H-11, H-15**), 7.53 (1H, dddd, *J* = 8.6, 6.9,1.3, 1.3, **H-13**), 7.46-7.42 (2H, m, **H-12, H-14**), 7.28-7.26 (4H, m, **H-5, H-9, H-6, H-8**), 7.23-7.17 (1H, m, **H-7**), 4.20 (1H, ddd, *J* = 14.1, 8.9, 5.0, **H-3**), 3.87 (1H, d, *J* = 9.4 Hz, **H-2**), 3.73 (3H, s, **H-18**), 3.57 (1H, dd, *J* = 16.9, 4.9 Hz, **H-16a**), 3.50 (3H, s, **H-20**), 3.48 (1 H, dd, *J* = 16.9, 8.7 Hz, **H-16b**).

^{13}C NMR (100 MHz, $CDCl_3$) δ (ppm): 197.6 (**C-1**), 168.9 (**C-17**), 168.3 (**C-19**), 140.6 (**C-4**), 136.9 (**C-10**), 133.2 (**C-11**), 128.6 (x2) (**C-5,C-9**), 128.2 (x2) (**C-6,C-8**), 127.3 (**C-15**), 57.4 (**C-16**), 52.8 (**C-18**), 52.5 (**C-20**), 42.4 (**C-2**), 40.9 (**C-3**).

IR (neat, cm^{-1}): 3667, 2989, 2958, 2901, 1728, 1681, 1234, 1159.

2. NMR Spectra

2.1. dimethyl 2-(3-oxocyclopentyl)malonate

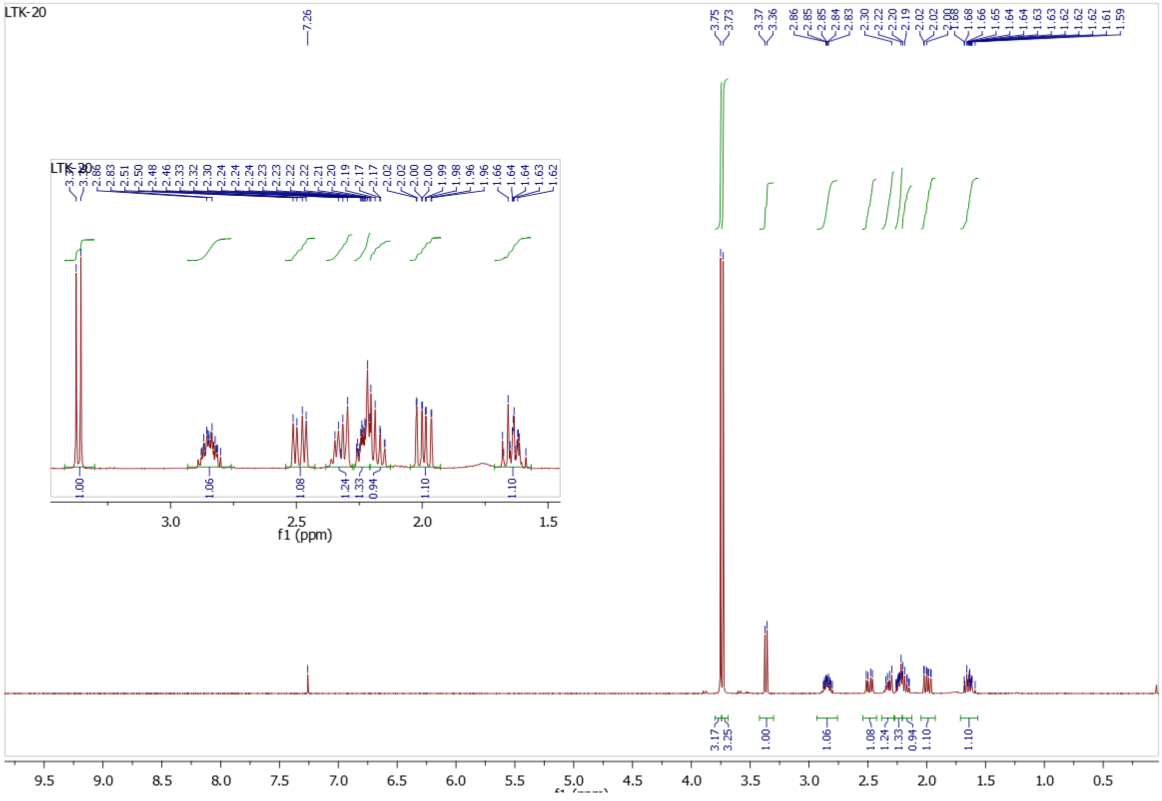

Figure S1. ^{1}H NMR (400 MHz) spectrum of dimethyl 2-(3-oxocyclopentyl)malonate

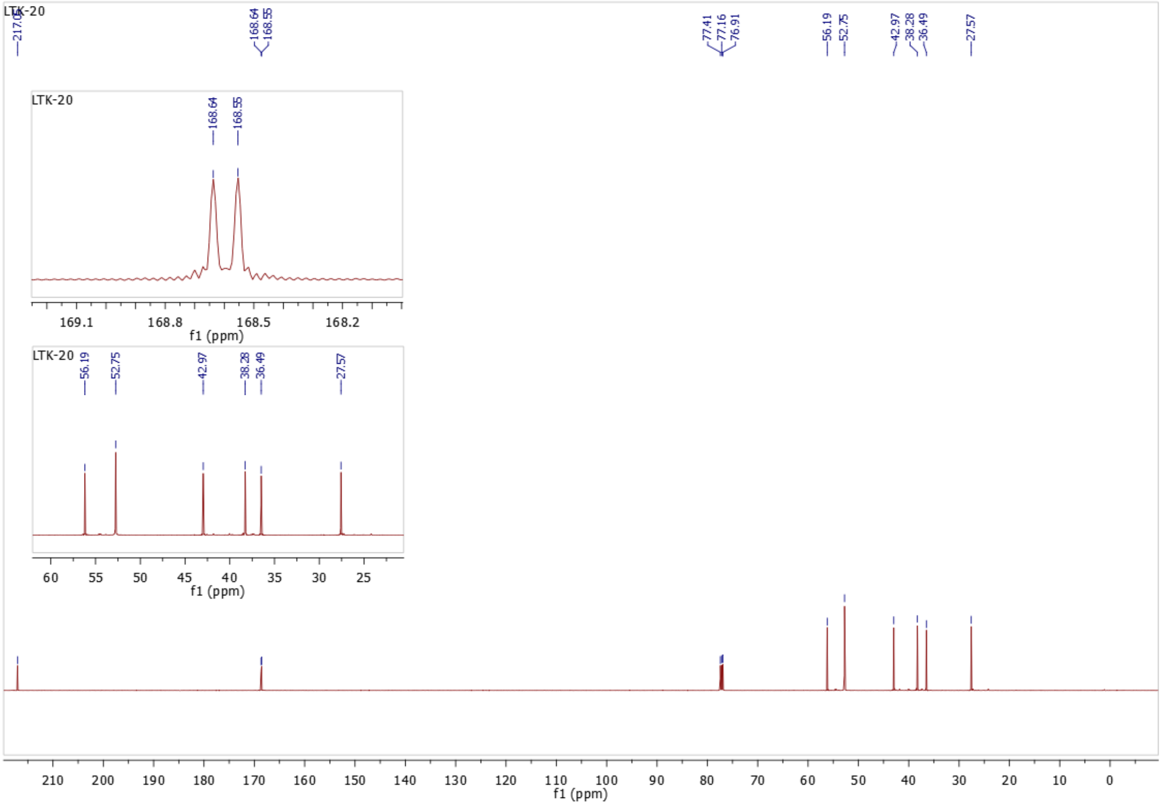

Figure S2. ^{13}C NMR (100 MHz) of dimethyl 2-(3-oxocyclopentyl)malonate

2.2. dimethyl 2-(3-oxocyclohexyl)malonate

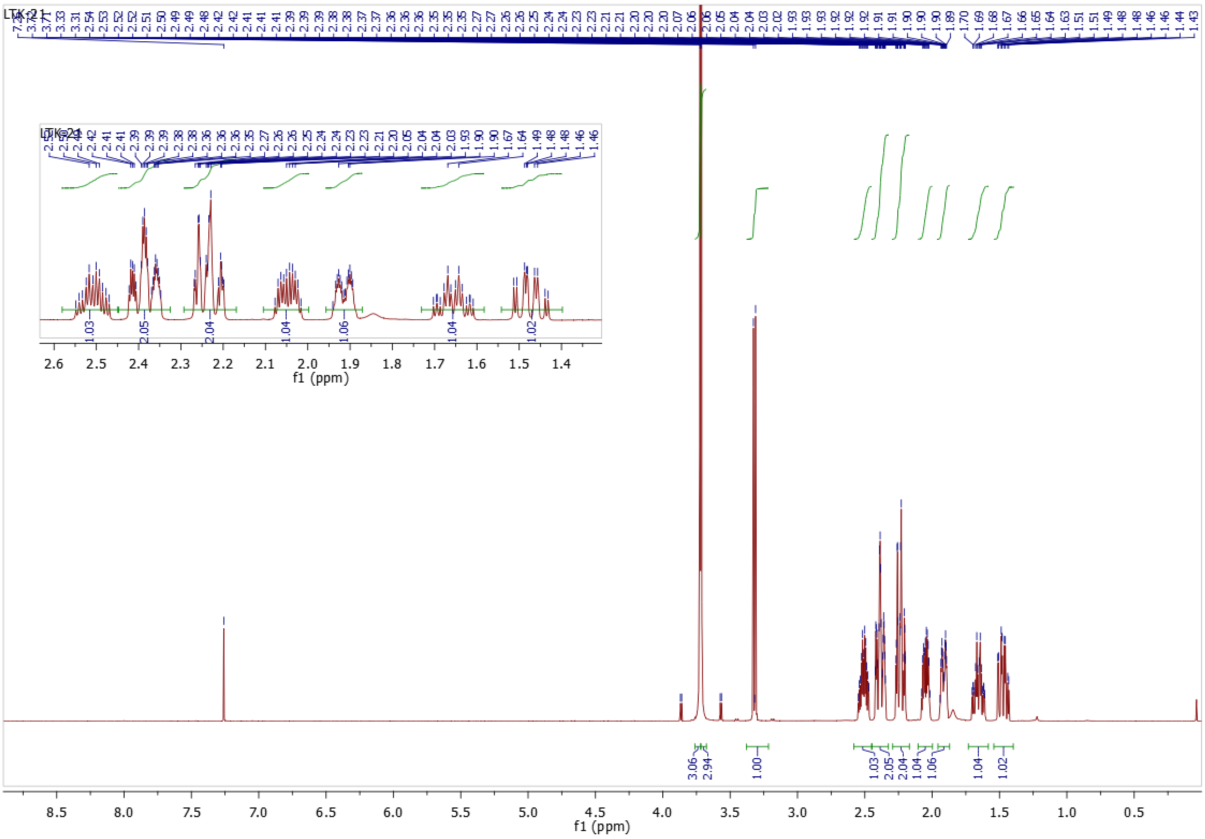

Figure S3. ^{1}H NMR (400 MHz) spectrum of dimethyl 2-(3-oxocyclohexyl)malonate

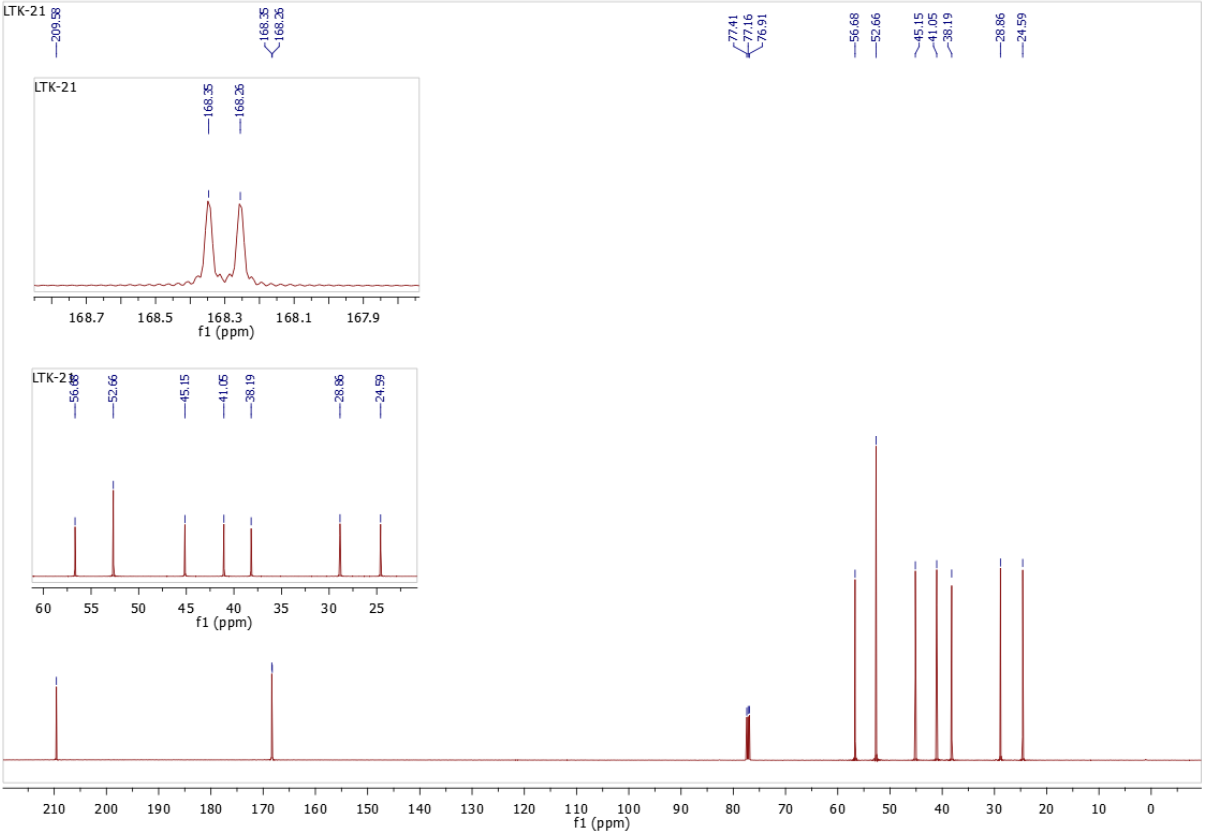

Figure S4. ^{13}C NMR (100 MHz) spectrum of dimethyl 2-(3-oxocyclohexyl)malonate

2.3. dimethyl 2-(3-oxo-1,3-diphenylpropyl)malonate

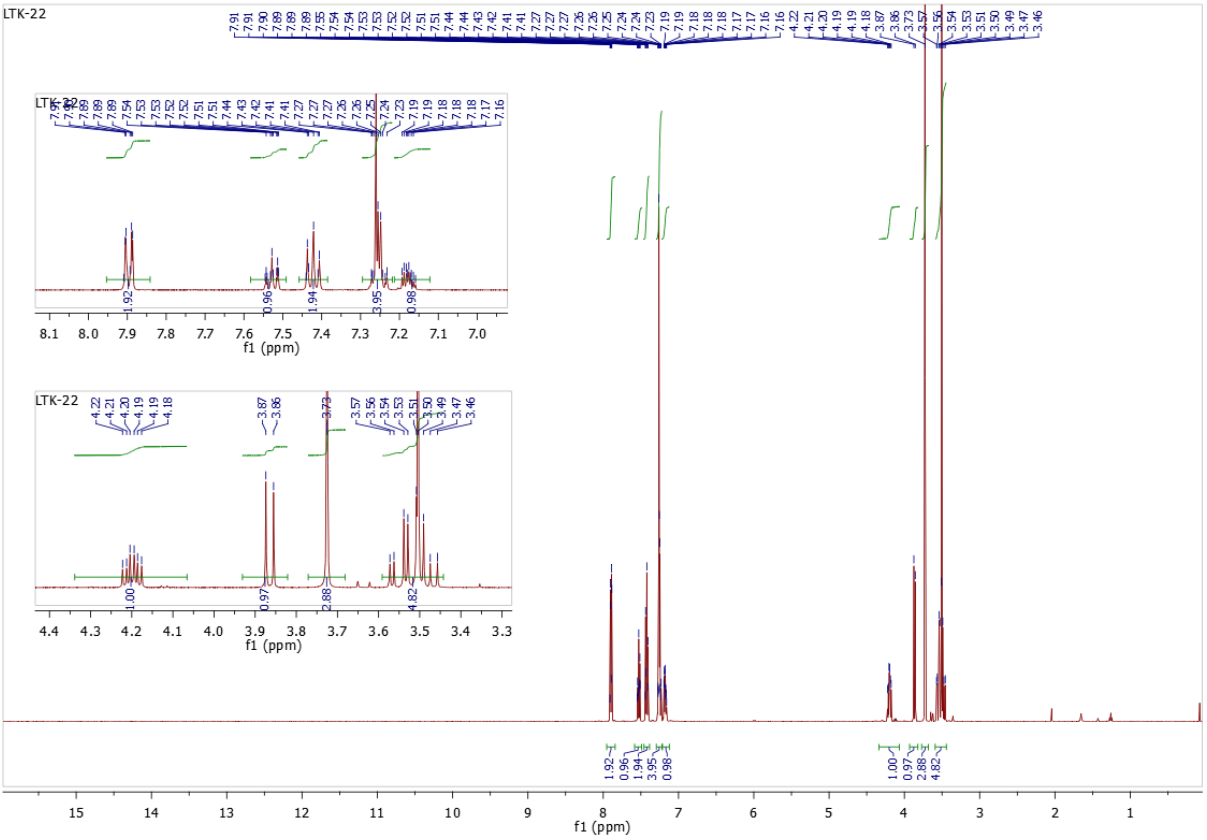

Figure S5. ^{1}H NMR (400 MHz) spectrum of dimethyl 2-(3-oxo-1,3-diphenylpropyl)malonate

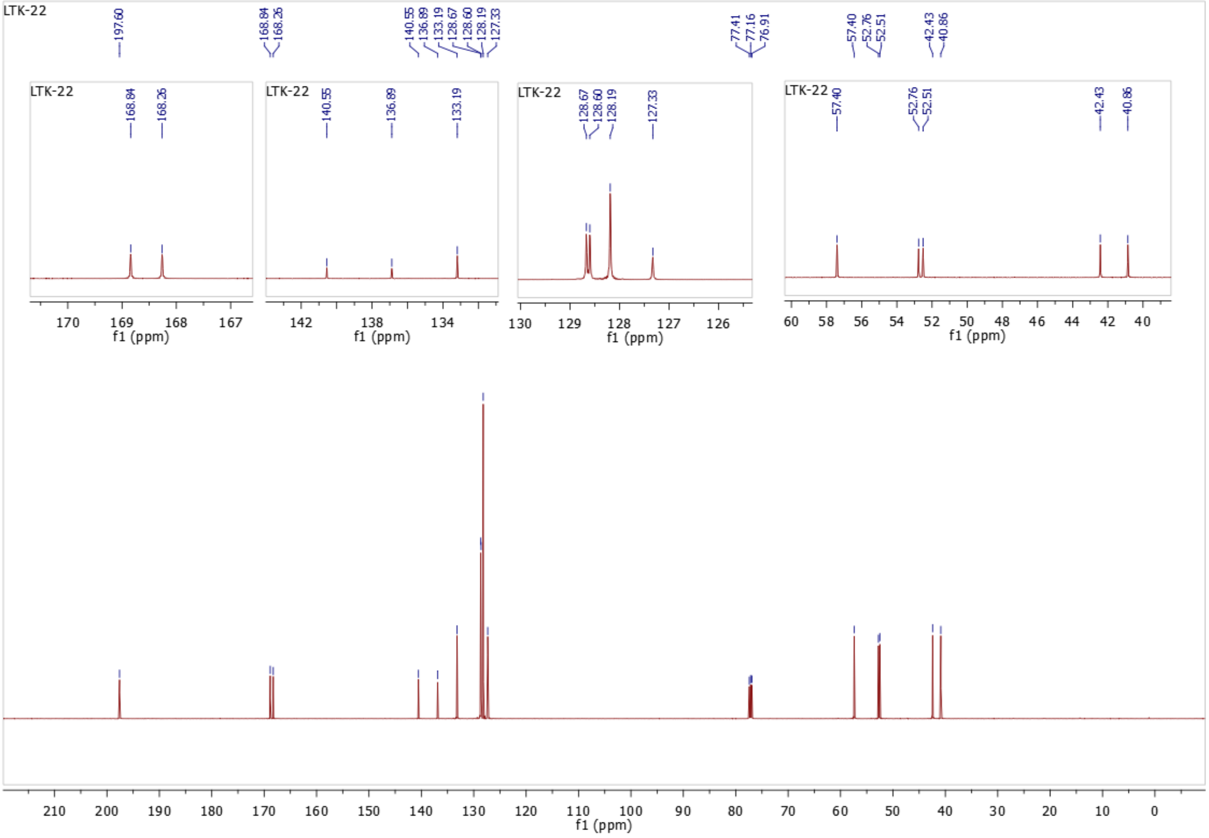

Figure S6. ^{13}C NMR (100 MHz) spectrum of dimethyl 2-(3-oxocyclopentyl)malonate

3. IR Spectra

3.1. dimethyl 2-(3-oxocyclopentyl)malonate

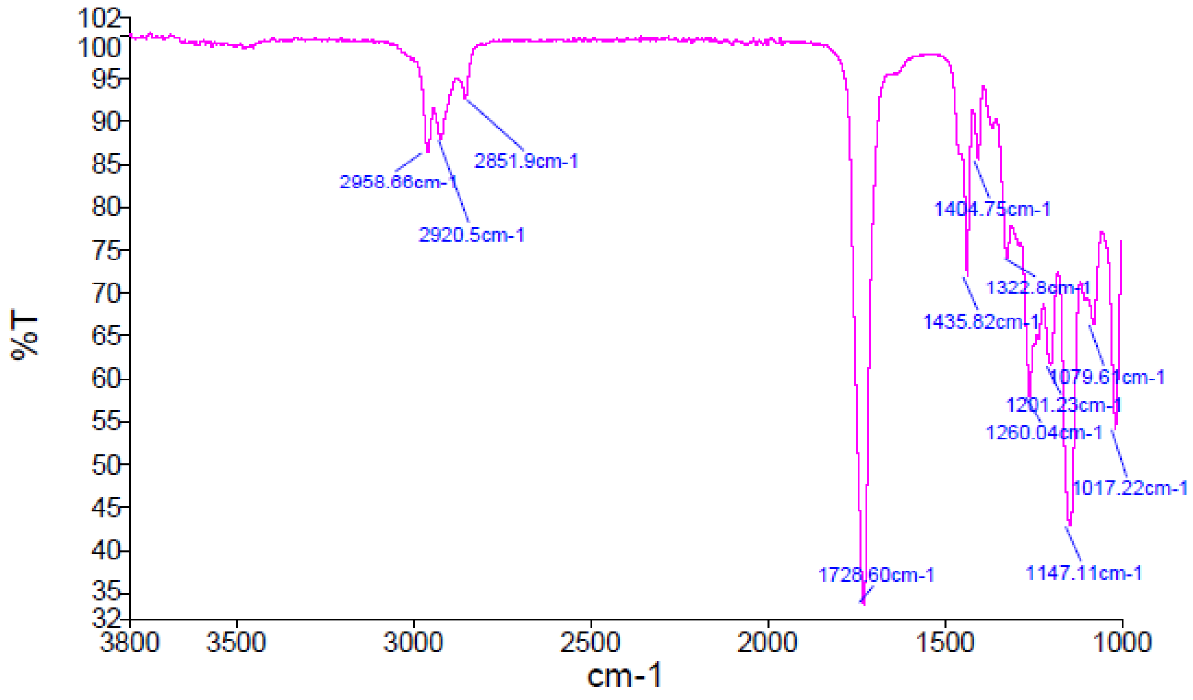

Figure S7. IR spectrum of dimethyl 2-(3-oxocyclopentyl)malonate

3.2. dimethyl 2-(3-oxocyclohexyl)malonate

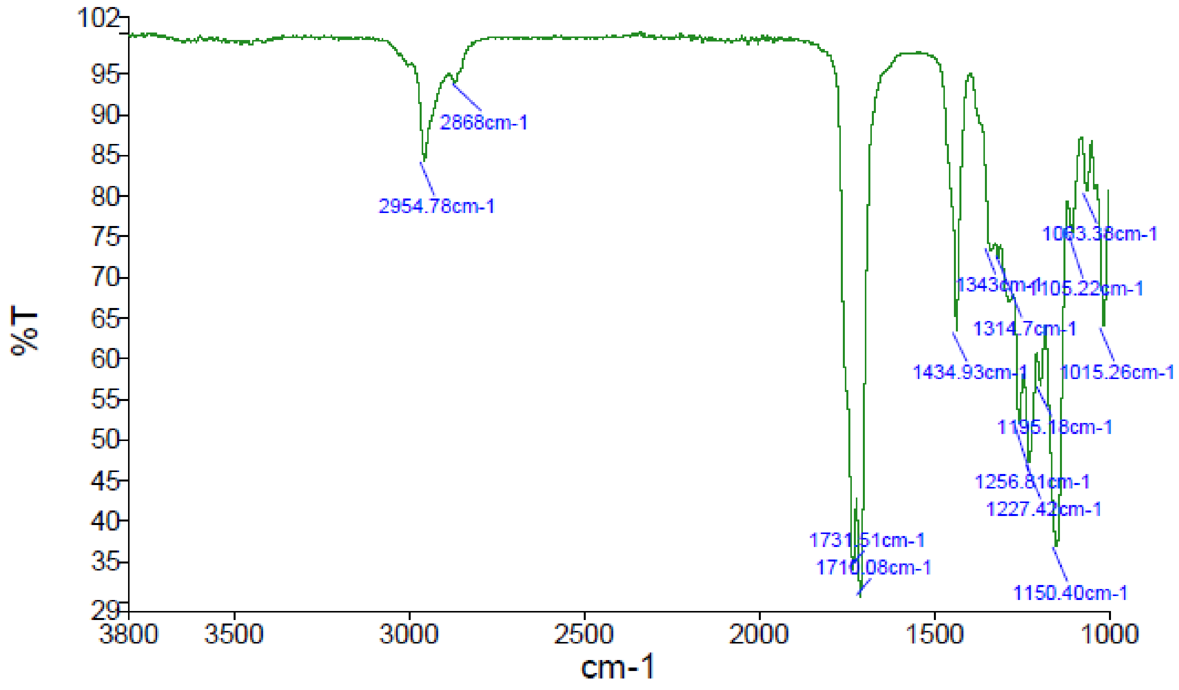

Figure S8. IR spectrum of dimethyl 2-(3-oxocyclohexyl)malonate

3.3. dimethyl 2-(3-oxo-1,3-diphenylpropyl)malonate

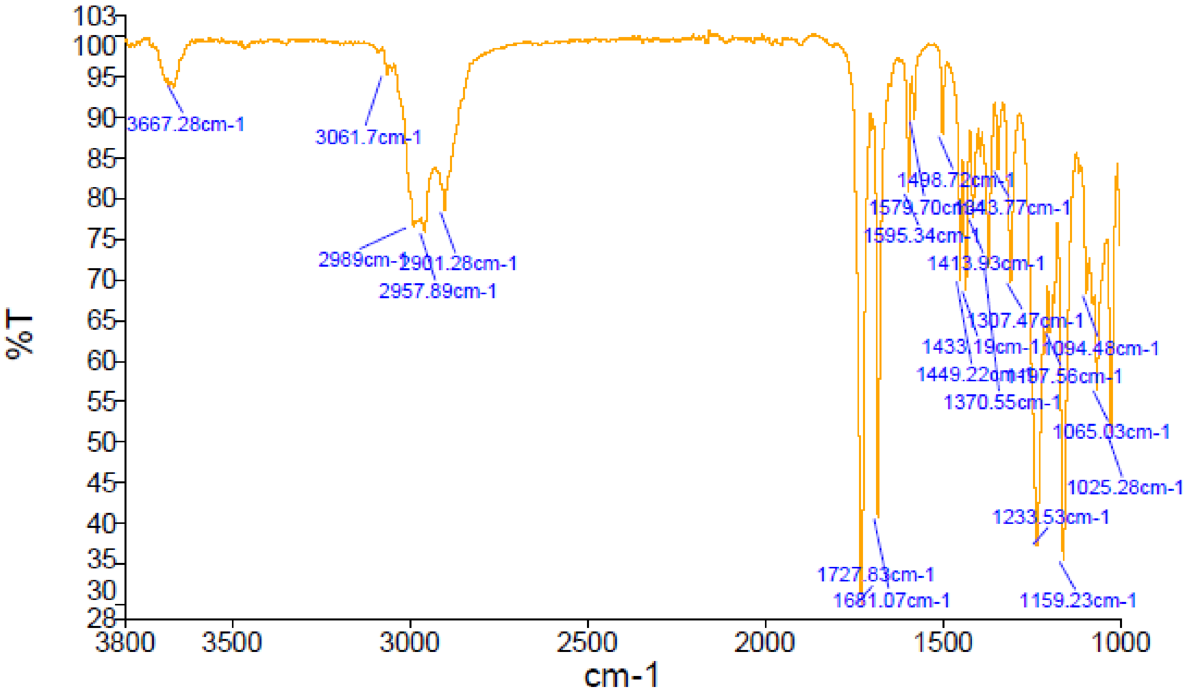

Figure S9. IR spectrum of dimethyl 2-(3-oxo-1,3-diphenylpropyl)malonate

molecules

4. ESBD Images

FSD Mixed Image 4

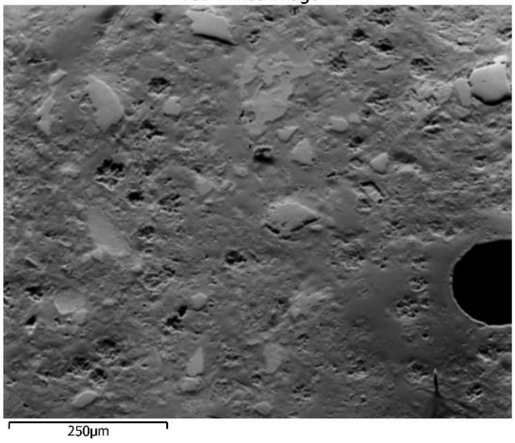

Table S1. ESBD Map Settings Table

Accelerating Voltage	20.00 kV
Specimen Tilt (degrees)	70.00 °
Hit Rate	14.87 %
Speed of Acquisition	39.46 Hz

Table S2. Phase for acquisition

Phase	a	b	c	Alpha	Beta	Gamma	Space Group	Database
Calcite	4.99 Å	4.99 Å	17.06 Å	90.00 °	90.00 °	120.00 °	0	HKL
Quartz	4.91 Å	4.91 Å	5.50 Å	90.00 °	90.00 °	120.00 °	0	CHANNELPhaseDatabase
K_Feldspar	8.57 Å	12.96 Å	7.22 Å	90.66 °	115.93 °	87.62 °	0	CHANNELPhaseDatabase
Dolomite	4.78 Å	4.78 Å	15.73 Å	90.00 °	90.00 °	120.00 °	148	ICSD
Calcite	4.99 Å	4.99 Å	17.06 Å	90.00 °	90.00 °	120.00 °	167	ICSD
Oxyapatite	9.43 Å	9.43 Å	6.88 Å	90.00 °	90.00 °	120.00 °	174	ICSD
Tremolite	9.86 Å	18.12 Å	5.29 Å	90.00 °	104.57 °	90.00 °	12	ICSD
Low albite	8.14 Å	12.78 Å	7.16 Å	94.19 °	116.61 °	87.68 °	0	CHANNELPhaseDatabase

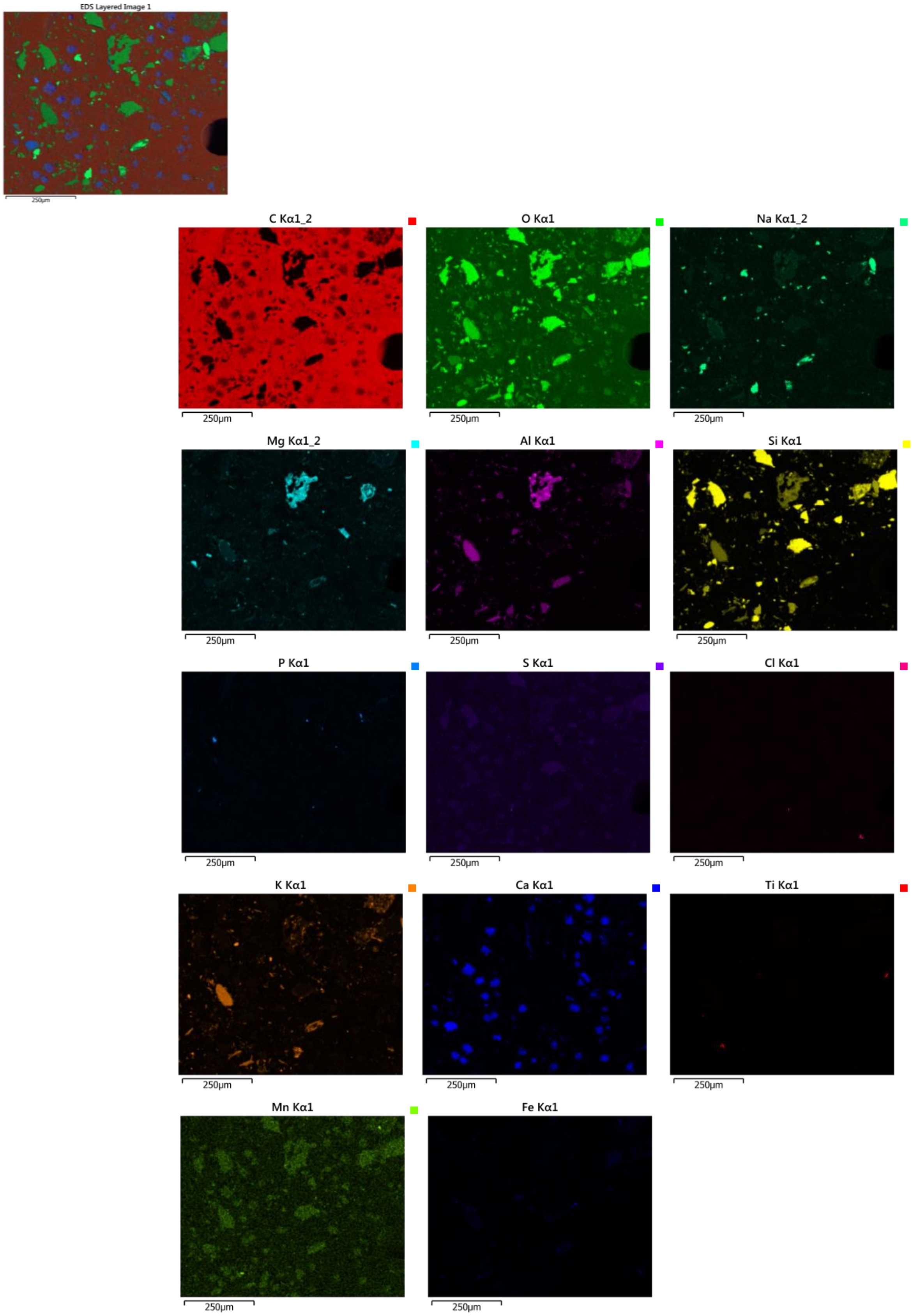

Figure S10. EDS Layered Images

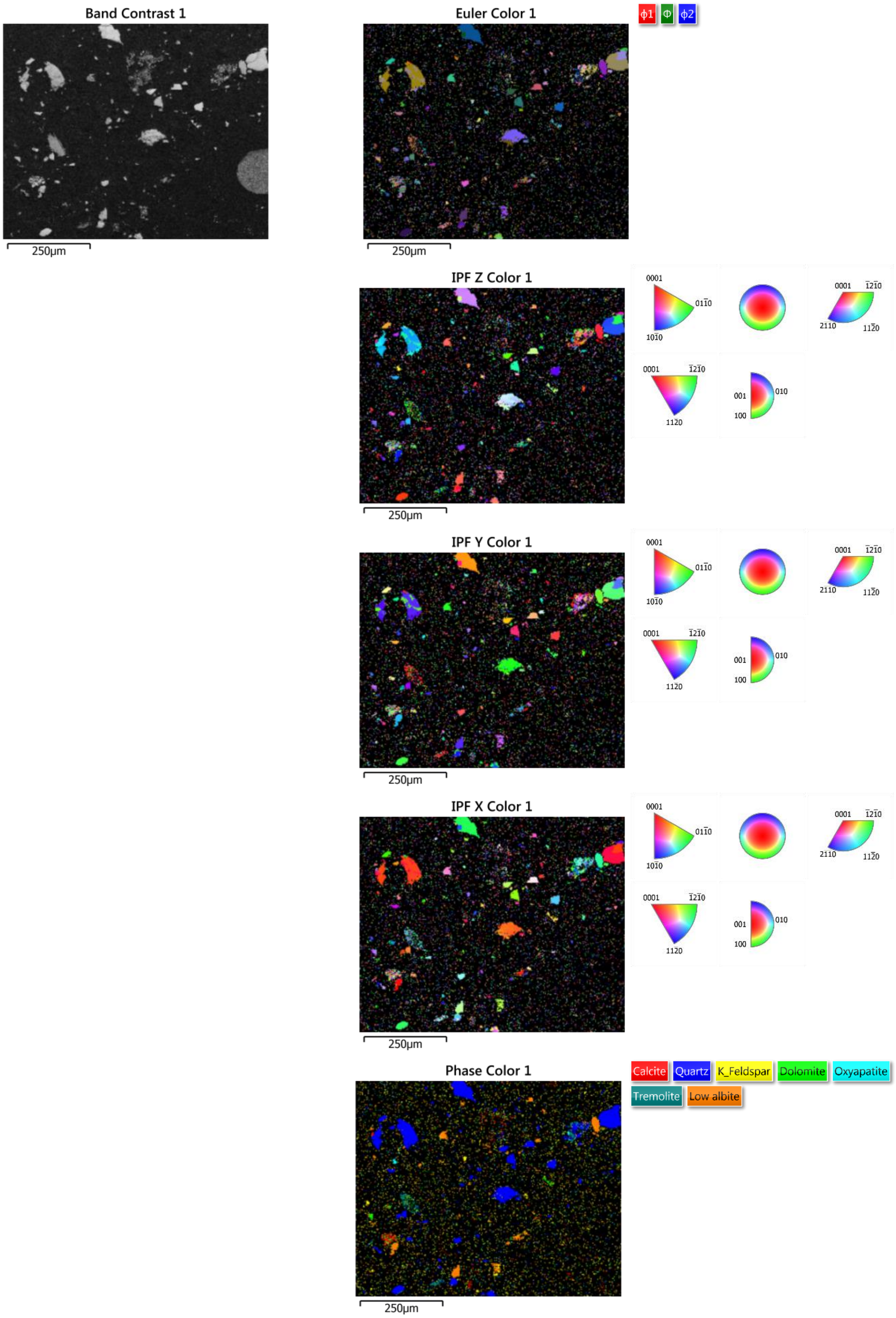

Figure S11. Phase coloration

Table S3 : Phase fraction

Phase Name	Phase Fraction (%)	Phase Count	Mean Band Contrast	Standard Deviation Band Contrast	Min Band Contrast	Max Band Contrast	Mean MAD	Standard Deviation MAD	Min MAD	Max MAD
Calcite	0.56	475	101.03	41.35	25.00	211.00	0.93	0.22	0.39	1.99
Quartz	4.73	3991	131.43	41.37	23.00	200.00	0.74	0.28	0.21	1.98
K_Feldspar	4.01	3384	45.25	18.08	23.00	154.00	1.26	0.28	0.34	2.00
Dolomite	0.91	769	64.40	39.51	24.00	196.00	1.18	0.34	0.31	1.99
Oxyapatite	0.35	299	54.99	31.69	25.00	193.00	1.23	0.31	0.39	2.00
Tremolite	1.65	1393	55.07	28.35	23.00	174.00	1.18	0.31	0.41	1.99
Low albite	2.65	2238	68.95	40.70	24.00	169.00	1.12	0.36	0.35	2.00
Zero Solutions	85.13	71815	42.37	12.46	0.00	177.00				

5. XRPD analysis

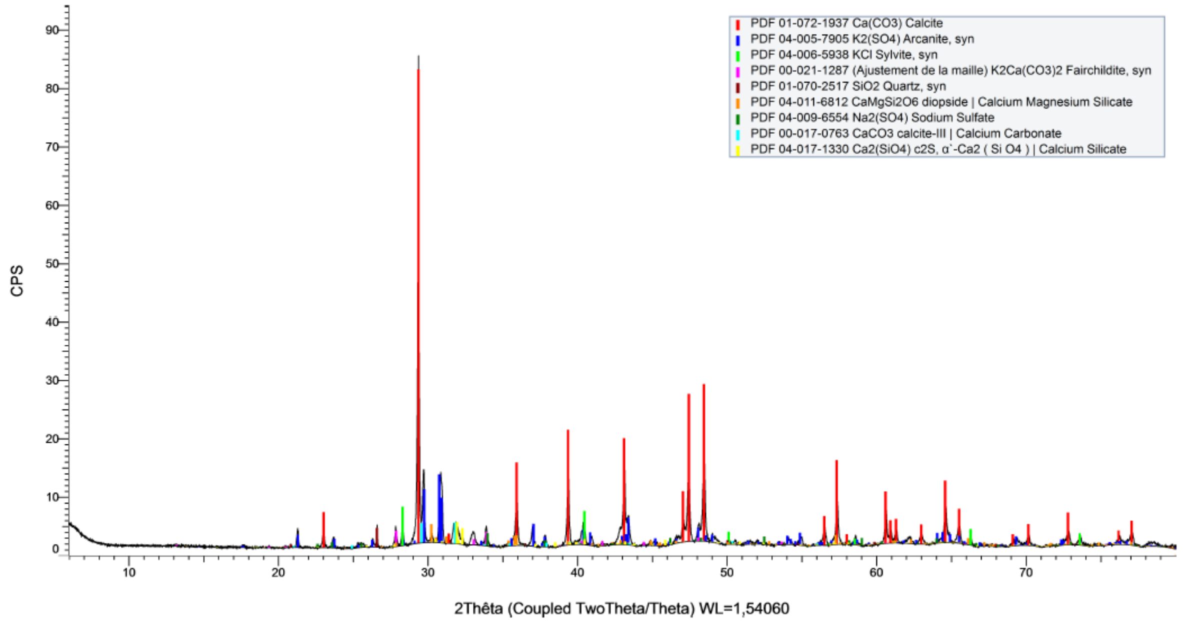

Figure S12 : XRPD analysis of Eco-base-Fj

6. Ionic chromatography

1.0018 g of Eco-base-Fj and water (10 mL) was stirred during 2 h. The solid state of Eco-base-Fj was centrifugated and the liquid state was analyzed.

Table S4. Ionic chromatography of Eco-base-Fj

**$[X^{n-}]$**	**tr (min)**	**$[X^{n-}]$ (g/L)**	**$[X^{n-}]$ (mg/g of Eco-base-Fj)**	**$[X^{n-}]$ (mmol/g of Eco-base-Fj)**	**$[K_nX]$ (mg/g of Eco-base-Fj)**
**$[Cl^-]$**	8.9	7.967	79.5	2.24	163
**$[SO_4^{2-}]$**	22.6	6.838	68.3	0.71	124
**$[CO_3^{2-}]$**	18.1	4.377	37.8	0.63	87
